# *Lavandula austroapennina* (Lamiaceae): Getting Insights into Bioactive Polyphenols of a Rare Italian Endemic Vascular Plant

**DOI:** 10.3390/ijms24098038

**Published:** 2023-04-28

**Authors:** Claudia Gravina, Marialuisa Formato, Simona Piccolella, Marika Fiorentino, Adriano Stinca, Severina Pacifico, Assunta Esposito

**Affiliations:** Department of Environmental, Biological and Pharmaceutical Sciences and Technologies, University of Campania ‘Luigi Vanvitelli’, Via Vivaldi 43, 81100 Caserta, Italy; claudia.gravina@unicampania.it (C.G.); marialuisa.formato@unicampania.it (M.F.); simona.piccolella@unicampania.it (S.P.); marika.fiorentino@unicampania.it (M.F.); adriano.stinca@unicampania.it (A.S.); assunta.esposito@unicampania.it (A.E.)

**Keywords:** *Lavandula austroapennina*, Cilento, Vallo di Diano and Alburni National Park, polyphenols, UHPLC-Q*q*TOF-MS/MS, HaCaT cell line, antioxidant activity, cytotoxicity, wound-healing property

## Abstract

*Lavandula austroapennina* N.G. Passal., Tundis and Upon has recently been described as a new species endemic to the southern Apennines (Italy). Locally, this species has a long ethnobotanical tradition of use for curative and decoration purposes and has been the protagonist of a flourishing essential oil production chain. Currently, while this tradition has long since ended, attention to the species is necessary, with a view to enhancing marginal and rural areas, as a recovery of a precious resource to (i) get insights into its (poly)phenolic fraction and (ii) address new and innovative uses of all its organs in various application fields (e.g., cosmeceutical sector). Therefore, after field sampling and dissection of its organs (i.e., corolla, calyx, leaf, stem and root), the latter, previously deterpenated and defatted, were subjected to accelerated ultrasound extraction and the related alcoholic extracts were obtained. Chemical composition, explored by UHPLC-Q*q*TOF-MS/MS, and the following multivariate data analysis showed that the hydroxycinnamoyl derivatives are abundant in the leaf, stem and root, while flavonoids are more present in corolla and calyx. In particular, coumaroyl flavonoids with glyconic portion containing also hexuronyl moieties differentiated corolla organ, while yunnaneic acid D isomers and esculin distinguished root. When antiradical and reducing properties were evaluated (by means of ABTS, DPPH and PFRAP tests), a similar clustering of organs was achieved and the marked antioxidant efficacy of leaf, stem and root extracts was found. Thus, following cytotoxicity screening by MTT test on HaCaT keratinocytes, the protective effects of the organ extracts were assessed by wound closure observed after the scratch test. In addition, the extracts from corolla, leaf and stem were particularly active at low doses inducing rapid wound closure on HaCaT cells at a concentration of 1 μg/mL. The diversity in (poly)phenols of each organ and the promising bioactivity preliminarily assessed suggest further investigation to be carried out to fully recover and valorize this precious endemic vascular plant.

## 1. Introduction

Since ancient times, medicinal–aromatic plants (MAPs) have been used to maintain health and to prevent and treat disease. Nowadays, they are attracting considerable interest as potential sources of bioactive chemicals [1]. MAPs-derived products have become a new trend and more and more people are using them, especially in the growing international market of plant-based products, including cosmetics, spices and health remedies [2].

**Figure 1:**
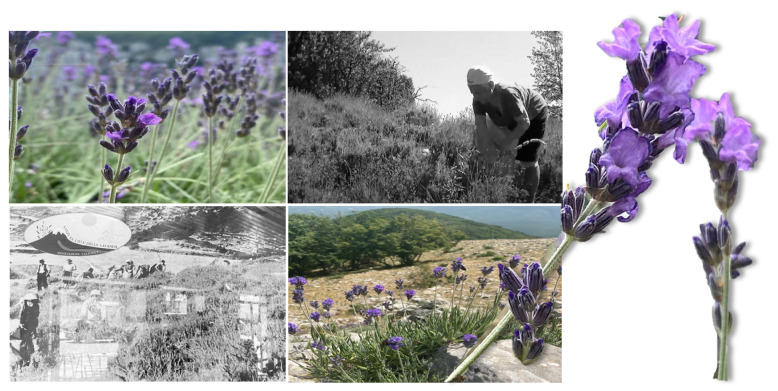
*Lavandula austroapennina*: pictures of habitat and old ethnobotanical uses of “*spicaddossa*”. Source: “Sanza Città della lavanda” cultural Association and Rofrano Proloco https://www.youtube.com/watch?v=txGsa4qmCR4 (accessed on 1 March 2023).

Therefore, the use of MAPs shows no sign of decreasing, and according to the World Health Organization (WHO), healthy-plant-based products are currently used by most of the world population (~80%) and among these are 100 million Europeans [3]. Indeed, there are over 1300 native medicinal plants in Europe [4], and the European Plant Conservation Strategy (EPCS) states that 90% of MAPs native to Europe are still harvested from the wild. The collection and use of medicinal plants have an ancient tradition in these areas, while recently ethnobotanical and phytochemical studies aimed at gathering knowledge of the plant heritage for exploring new resources and also preserving local populations’ traditional culture [4,5,6]. This is especially important for species in a restricted range and limited to specific geographical areas (endemic species), where these floristic elements could provide a profitable medical and economic value for local communities [7]. Among MAPs, the Lamiaceae family includes a large number of species commonly used for culinary purposes as aroma and/or flavor enhancers [8,9]. In this family, the genus *Lavandula* stands out for its traditional application in the treatment of depression, headache, stress, migraine and diabetes [10]. Several species of *Lavandula* (e.g., *L. angustifolia* Mill., *L. latifolia* Medik., *L. pedunculata* (Mill.) Cav., *L. stoechas* L. and *L. × intermedia* Emeric ex Loisel) have been cultivated since early 20th century for the extraction of their essential oils (EOs) used in perfumery, cosmetics, food processing and aromatherapy [11]. Only recently, the awareness of the possibility of recovering bioactive (poly)phenols from different species belonging to this genus has been increasing. These compounds, broadly differing in their chemical features, are well-known as antioxidants able to neutralize free radicals, thus preventing cell and tissue damage and the onset of pathological diseases [9]. The antioxidant and anti-inflammatory properties of polar extracts of *L. angustifolia*, *L. stoechas*, *L. dentata* and *L. pedunculata* [12,13,14] were recently investigated, thus also enhancing the economic interest in these species, mainly in the widely cultivated *L. angustifolia*. The latter, commonly used as an ornamental plant and known for the high quality of its EOs, consists of numerous cultivars [15] with a broad distribution from Spain through France to Italy, where it occurs as a mountain species at altitudes above 1500 m asl. The enormous natural range of variation of *L. angustifolia* allowed Upson and Andrews [16] to observe disjunct populations of *L. angustifolia* subsp. *angustifolia* in southern Italy. Thus, further morphological, genetic and phytochemical analyses of EOs [17,18] led to describe populations of *L. angustifolia* subsp. *angustifolia* from south-eastern Italy as a new Italian endemic species: *Lavandula austroapennina* N.G. Passal., Tundis and Upson (Figure 1). This species, categorized as least concern (LC) in the IUCN Red List of Italian Flora [19], is restricted to rocky calcareous habitats from 900 to 1750 m asl, in the Southern Apennines phytogeographic area [17].

“*Spicaddossa*” is the local name, as it is popularly used as a remedy, rubbing the leaves, for disinfectant and soothing purposes [20]. Indeed, until the 1960s, the wild lavender from Monte Cervati in the Municipality of Sanza (Cilento, Vallo di Diano and Alburni National Park—Campania Region) was the main actor of the perfume supply chain, so that the plant locally collected served for extracting valuable essential oils, which underwent final processing when exported to France. However, nowadays this local tradition has abruptly stopped [21]. EOs extracted from plants collected from Monte Pollino (Pollino Global Geopark—Calabria Region) [17,18] were phytochemically investigated, while, as far as we know, no data are reported for their (poly)phenolic profile and bioactivity.

In light of the above, the present work aims to increase the occurrence of (poly)phenol compounds in *L. austroapennina* organs and to evaluate their antioxidant activity and healing efficacy. To this aim, after harvesting, the plant was dissected into corolla, calyx, stem, leaf and root. Then, a sequential ultrasound-assisted maceration (UAM) was performed using first *n*-hexane as an extractive solvent, for matrix defatting purposes, and then methanol, which allowed us to effectively recover (poly)phenol compounds, due to their polarity and solubility features. The alcoholic extract was chemically profiled by ultra-high liquid chromatography with high-resolution mass spectrometry (UHPLC-HR-MS/MS). The antiradical activity was evaluated by DPPH^•^ and ABTS^•+^ scavenging assays, while the reducing power of ferric ions was evaluated by the PFRAP test. Cytotoxicity was assessed on HaCaT cell lines at different treatment times, and healing activity was evaluated by means of the scratch test.

## 2. Results and Discussion

### 2.1. Chemical Investigation of L. austroapennina Alcoholic Extracts

**Figure 2:**
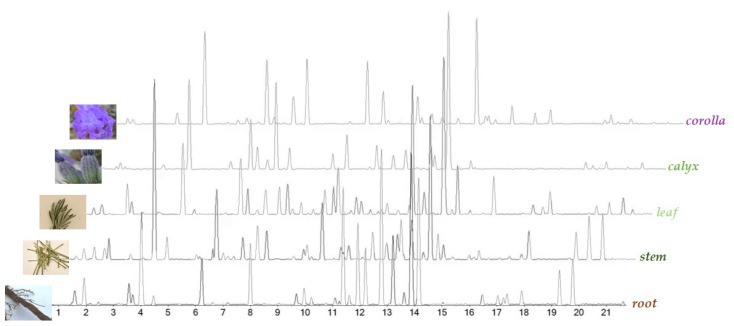
Base Peak Chromatograms (BPCs) of *L. austroapennina* alcoholic extracts from its organs.

UHPLC-ESI-Q*q*TOF analysis was carried out on all the alcoholic extracts, in order to achieve their chemical profiles. The Total Ion Chromatograms (TICs), reported in Figure 2, clearly show the peculiarity of each plant organ in terms of chemical composition. The heatmap and multivariate analysis confirmed this evidence (for details please see below—Section 2.2).

Based on high-resolution tandem mass spectrometry data, 67 compounds were tentatively identified, distinguishable into subclasses (phenylpropenoic and phenylpropanoic acid derivatives, and flavonoids; Table 1, Table 2 and Table 3), and discussed separately.

#### 2.1.1. Phenylpropenoic Acid Derivatives

Different hydroxycinnamic acids with *p*-coumaroyl, caffeoyl and feruloyl base skeletons were tentatively identified (Table 1), some of which have been recently reported in oil-exhausted aerial part biomasses of *L. angustifolia* and *L. × intermedia* cv. “Grosso” [22].

Compound **3** was tentatively identified as caftaric acid, due to the fragment ion at *m/z* 149.0094, corresponding to the deprotonated tartaric acid. It was previously found in *O. basilicum* L., *O. vulgare* L. and *T. vulgaris* L. aerial parts by UHPLC-MS/MS analyses [23]. Fertaric (feruloyl tartaric) acid was also recognized (**7**).

Furthermore, compounds **11** and **12** with the [M-H]^−^ ion at *m/z* 295.0462(3) were likely to be two isomers of caffeoylmalic acid.

Compounds **8** and **16** with the [M-H]^−^ ion at *m/z* 341.0872(83) were putatively caffeoyl hexosides, which were observed in *Lavandula x intermedia* Emeric (ex Loisel) waste [24] as well as in *L. pedunculata* (Mill.) Cav. flowering stems with inflorescence [25]. Compound **5**, likely esculin, was mainly abundant in the root extract.

The other hydroxycinnamic acids were also found as glycosides. Indeed, *p*-coumaric acid dihexoside (**9**) was tentatively identified. The neutral loss of a dehydrated di-hexose (324.10 Da) from the deprotonated molecular ion provided fragment ions at *m/z* 163.0394 (*p*-coumarate ion) and its decarboxylated ion at *m/z* 119.0501. Accordingly, metabolites **6** and **13** were tentatively identified as *p*-coumaric acid hexosides. *Lavandula x intermedia* Emeric (ex Loisel) methanol waste extracts were rich in *p*-coumaroyl derivatives [24]. Compounds **10** and **18** were feruloyl hexoses, which occurred together with the dihexosyl derivative **14**. The deprotonated aglycone ion appeared at *m/z* 193.05 and, in line with the other hydroxycinnamates, lost CO_2_ generating the base peak at *m/z* 149.06. It is worthy of note that the Lamiaceae family is a rich source of ferulic acid and its derivatives, which were found in the ethanolic extracts of *L. angustifolia* Miller, *Teucrium* spp. and *Micromeria thymifolia* (Scop.) Fritsch. [26]. In particular, within the *Lavandula* genus, the 4-*O-β*-d-glucopyranosyl derivative of ferulic acid was isolated from *L. angustifolia* subsp. *angustifolia* (=*L. spica* L.) flowers [27].

Compounds **61** and **63** were putatively recognized as two isomers of (dihydroxyphenyl)ethenyl-3-(dihydroxyphenyl)prop-2-enoate, whose assigned trivial names are nepetoidin A and B (Figure S1), respectively, based on literature data [28].

Finally, a tri-*p*-coumaroylspermidine (**66**; *m/z* 582.2626), recently described for *Salvia officinalis*, *S. lavandulifolia*, *S. sclarea*, *S. cadmica*, *S. nemorosa*, *S. tomentosa* and *Lavandula augustifolia* Mill. [29], was also detected. The loss of one (or two) hydroxystyrene unit (120.05 Da) gave rise to fragment ions at *m/z* 462.2058 and 342.1466, respectively, whereas the loss of dehydrated *p*-coumaric acid provided fragment ion at *m/z* 436.2262 [30].

#### 2.1.2. Phenylpropanoic Acid Derivatives

Compounds **1** and **2** were tentatively 8-hydroxy-dihydrocaffeic acid (danshensu) and its hexosyl derivative, respectively (Table 2). In fact, TOF-MS/MS spectra of compound **2** exhibited the neutral loss of 162.05 Da (hexose moiety) providing fragment ion at *m/z* 197.0456, from which the fragment ion at *m/z* 179.0344(57) was generated by H_2_O loss (Figure S2). Metabolite **4** was likely dihydrocaffeic acid, whereas the HR-MS/MS spectrum of compound **15** was in accordance with a dihydroferulic acid hexoside. In fact, following the loss of the saccharidic moiety, the aglycone ion at *m/z* 195.0661 generated the base peak at *m/z* 151.0766 (-CO_2_), which in turn underwent methyl radical loss to give the ion at *m/z* 136.0530.

**Figure 3:**
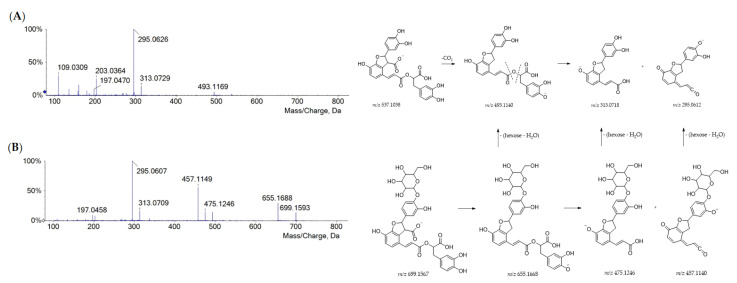
TOF-MS/MS spectra of compounds **27** (**A**) and **20** (**B**) with their putative main fragmentation patterns. Theoretical *m/z* values are reported below each structure.

Danshensu was also embedded in the molecular skeleton of rosmarinic acid and salvianolic acids. The salvianolic acids have been used in traditional Chinese medicine for the treatment of cardiovascular diseases for more than a thousand years [31]. Compared with other phenolic compounds, salvianolic acids have stronger antioxidant activity and other biological activities, such as hepatic and neural protection, and anticancer activity [32,33,34,35]. Herein, three salvianolic acids were recognized, eluting based on their decreasing polarity. Salvianolic acid B (**43**) is constituted of three danshensu and one caffeic acid units. From its [M-H]^−^ ion (at *m/z* 717.1487), the neutral loss of a danshensu moiety (or its dehydrated form) provided fragment ions at *m/z* 537.1062 (−180 Da) and 519.0953 (−198 Da), whereas one of the free carboxylic groups underwent decarboxylation, generating the product ion at *m/z* 673.1598 [25]. Further similar fragmentation pathways led to the base peak at *m/z* 295.0609 (Figure S3). Deprotonated compounds **57** (at *m/z* 491.0997) and **67** (at *m/z* 493.1163) were likely to be salvianolic acid C and salvianolic acid A, respectively (Figure S4). In both TOF-MS/MS spectra, the loss of 180.04 Da and then of 44 Da generated fragment ions at *m/z* 311.0563 and 267.0661 for salvianolic acid C and fragment ions at *m/z* 313.0711 and 269.0808 for salvianolic acid A. Based on the similar fragmentation pattern, compound **65** was tentatively identified as dihydrosalvianolic acid A. The [M-H]^−^ ion detected for metabolite **27** at *m/z* 537.1051 was in line with lithospermic acid A (Figure 3A).

As for salvianolic acid B, its occurrence was previously reported in Portuguese *Lavandula pedunculata* extracts [25]. Its hexoside was found in metabolite **20** (C_33_H_32_O_17_), whose hypothesized structure is depicted in Figure 3B, together with the TOF-MS/MS spectrum and fragmentation pathway.

Compound **35** was identified as rosmarinic acid hexoside, while rosmarinic acid was recognized in compound **41** with deprotonated molecular ion at *m/z* 359.0769. The isomer (**38**) of this latter was further detected. This compound is widespread in the Lamiaceae family, including the leaf ethanol extract of *Lavandula angustifolia* L. [36]. Health-related properties of rosmarinic acid have been deeply studied, so that a broad range of applications have gained attention, from the food sector to cosmetics [37,38,39,40].

**Figure 4:**
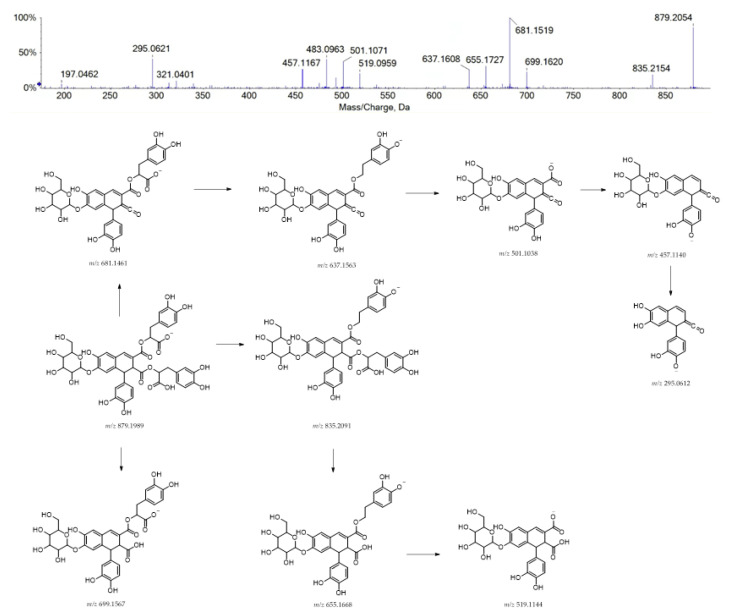
TOF-MS/MS spectrum of compound **40** and its hypothesized main fragmentation pattern. Theoretical *m/z* values are reported below each structure.

Finally, compound **40** was supposed to be structurally related to rosmarinic acid. It could be a hexosyl caffeic acid tetramer, also regarded as rosmarinic acid dimer (e.g., radbosiin), based on the [M-H]^−^ ion at *m/z* 879.1991 (Figure 4). This compound, isolated and identified from the stem of *Rabdosia japonica* for the first time, was recently characterized from *Origanum vulgare* [41]. To the best of our knowledge, this is the first report of its occurrence in the *Lavandula* genus.

Moreover, the TOF-MS/MS spectrum of metabolite **58** was in accordance with sinapoyl-hexosyl-rosmarinic acid, recently identified in *Salvia bulleyana* Diels aerial parts [42] and isolated for the first time from *Dracocephalum foetidum* Bunge, both belonging to Lamiaceae [43]. The loss of 368.10 Da (dehydrated sinapoyl-hexose) from the deprotonated molecular ion provided fragment ion at *m/z* 359.0785, whereas the loss of 198.06 Da (danshensu) and 180.04 (danshensu-H_2_O) provided fragment ions at *m/z* 529.1383 and 547.1479, respectively. From the latter, the loss of 224.06 Da (sinapic acid) led to the fragment ion at *m/z* 323.0772.

**Figure 5:**
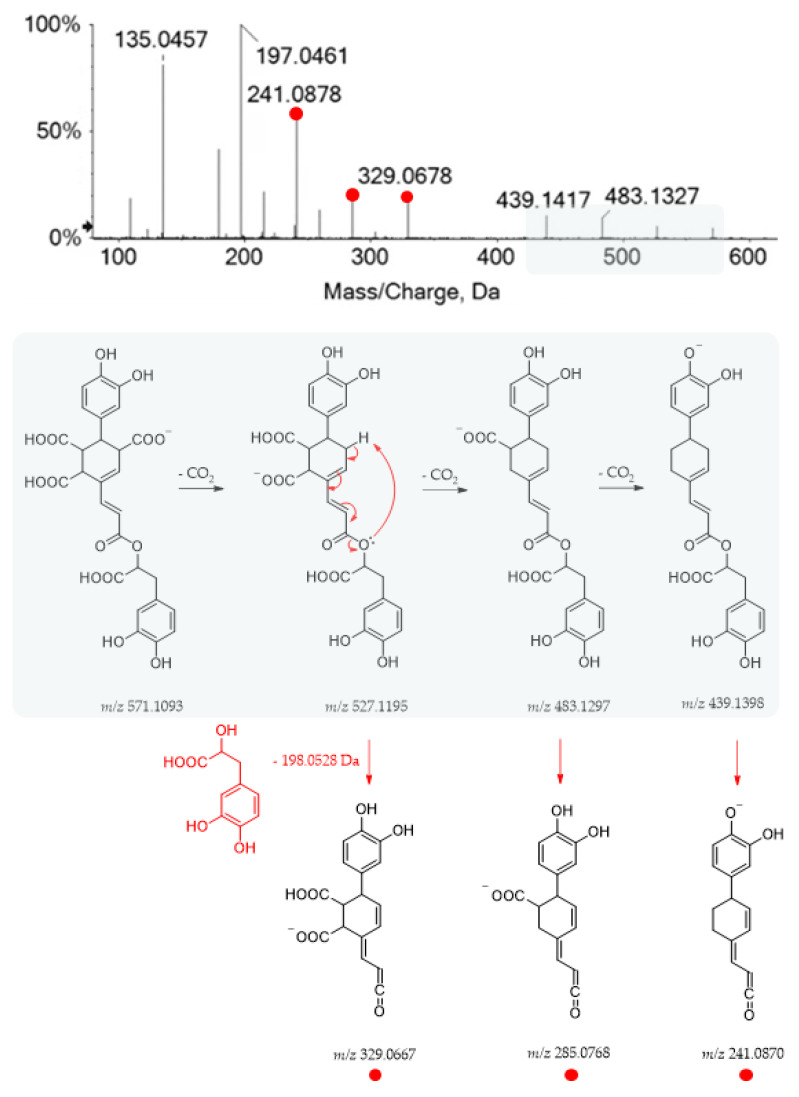
TOF-MS/MS spectrum of compound **19** and its hypothesized main fragmentation pattern. Theoretical *m/z* values are reported below each structure.

The TOF-MS and MS/MS spectra recorded for metabolite **19** suggested the occurrence of yunnaneic acid E, whose fragmentation pathway is reported in Figure 5.

Briefly, three decarboxylation reactions took place, each followed by the loss of the danshensu moiety (198.0528 Da), which appeared also as a base peak, further dissociating in the product ions, as previously described for compound **1**. It was present in a considerable amount in the aerial parts of different *Salvia* species (*S. blepharochlaena* Hedge and Hub., *S. euphratica* Montbret and Aucher, *S. verticillata* L. subsp. *amasiaca* Freyn and Bornm.) [44]. Based on this structure and related TOF-MS/MS spectrum, metabolite **48** was putatively identified as a derivative of yunnaneic acid E, formed through decarboxylative reduction. The deprotonated molecular ion was easily dehydrated to give the fragment ion at *m/z* 507.0953, characterized by an anhydride function. Then, it lost danshensu, following the mechanism previously described for yunnaneic acid E (Figure S5). Furthermore, compound **53**, which exhibited the [M-H]^−^ ion at *m/z* 523.0882, was likely a reduced derivative of compound **48**. Its putative structure and pivotal fragment ions are depicted in Figure S6, together with its HR-MS/MS spectrum. To the best of our knowledge, they have never been reported before in the literature.

Two isomers of yunnaneic acid F (**28** and **29**) were tentatively identified, as well as their putative dehydrated derivative (**51**) with [M-H]^−^ ion at *m/z* 579.1157.

Compounds **30**, **31** and **36** were identified as isomers of 6-(3-(1-carboxy-2-(3,4-dihydroxyphenyl)ethoxy)-3-oxoprop-1-en-1-yl)-3-(3,4-dihydroxyphenyl)-8-hydroxy-7-oxobicyclo [2.2.2]oct-5-ene-2-carboxylic acid (e.g., yunnaneic acid D isomers). From the precursor ion, a neutral loss of caffeic acid through a retro-Diels Alder reaction generated the fragment at *m/z* 359.08 (rosmarinic acid). As an alternative route, they fragmented in a concerted mechanism, losing the carboxylic group and danshensu, giving the product ion at *m/z* 297.0770(8). Thus, the latter could correspond to deprotonated 9-(3,4-dihydroxyphenyl)-7-hydroxy-1,6,7,8a-tetrahydro-1,6-methanonaphthalene-2,8-dione. This hypothesis is reported in Figure S7.

#### 2.1.3. Flavonoids

Flavonoids were mainly detected in calyx and corolla extracts, followed by leaf and stem ones. On the contrary, they were almost absent in root extract, in line with their role in response to plant biotic and abiotic stressors, which justify their abundance in vegetative and reproductive organs [45].

Herein, glycosylated derivatives of tricetin, luteolin and apigenin (which also occurred as aglycones—**56**, **60** and **50**, respectively) were tentatively identified (Table 3; Figure S8), while compounds **24** and **42** were myricetin and kaempferol hexosides, respectively (Figure S9).

Compounds **21**, **22**, **39**, **44**, **45**, **55**, **59** and **64** were apigenin derivatives. The TOF-MS/MS spectrum of compound **21** was in accordance with an apigenin di-hexuronide; considering that most commonly the hydroxyl group at C-5 position is involved in a H-bond with the carbonylic function, the two units of hexuronic acid are likely linked to positions 7 and 4′ [46]. They were lost as dehydrated form (176.03 Da), providing the fragment ions at *m/z* 445.0802 and 269.0463. In compound **22**, one hexuronic acid residue was substituted by a hexose. Accordingly, the molecular formula showed two H atoms instead of an oxygen, and the neutral losses leading to aglycone ions corresponded to 176 and 162 Da. Compound **39** differed from this latter for the presence of an acyl group linked to the hexose, identified as malonic acid. In fact, apart from a decarboxylation reaction giving the fragment at *m/z* 649.1464, the loss of the dehydrated malonyl moiety (C_3_H_2_O_3_; 86.0004 Da) likely led to compound **22** (at *m/z* 607.13). Thus, it was tentatively characterized as malonylhexosyl hexuronidyl apigenin.

The saccharidic units, whose cleavage led to metabolites **44** (at *m/z* 445.0794; C_21_H_18_O_11_), **45** (at *m/z* 431.0984), **55** and **59** (at *m/z* 473.1100/473.1098), were in accordance with a hexuronic acid, a hexose (−162.05 Da) and two acetyl-hexoses (−204.06 Da), respectively. Moreover, the [M−H]^−^ ion detected for metabolite **64** at *m/z* 577.1371 was likely attributed to apigenin *p*-coumaroyl-hexoside, identified by the neutral loss of 308.09 Da (*p*-coumaroylhexose-H_2_O). These apigenin derivatives have been already identified in the methanol extract of *Lavandula angustifolia* Mill. [47], *L. multifida* L. leaves [48], *L. coronopifolia* Poir. aerial parts and exhausted aerial parts of both *L. angustifolia* Mill. and *L. × intermedia* [22].

Luteolin hexuronyl-dihexoside (**17**), dihexuronide (**23**), hexuronyl-hexoside (**25**), hexuronides (**32** and **47**) and hexoside (**37**) were also recognized. The identification of the glyconic moieties followed the same neutral losses discussed before. Luteolin derivatives were depicted in methanolic extract of *L. multifida* leaves [48] and *L. × intermedia* cv. super aerial part waste [24]. The di-glycosylated flavonoid was already reported in the hydroalcoholic extract of *L. angustifolia* Mill. and *L. × intermedia* cv. super waste of aerial part [22], as well as in *L. dentata* and *L. stoechas* through a metabolomic approach [49]. The TOF-MS/MS spectrum of compound **52** (at *m/z* 533.0947) was in accordance with the malonyl ester of luteolin hexoside. In fact, the losses of CO_2_ (44 Da) from the acyl group and of 248.05 Da (dehydrated glucuronic acid + malonyl group) were observed. This compound was reported in an ethanolic extract from *L. angustifolia* leafy stalks and flowers [50]. Collision-induced fragmentation of metabolite **49** (at *m/z* 917.2357) allowed us to detect the presence of three hexosyl residues and a *p*-coumaroyl one on the aglycone skeleton, whereas in compound **46** (at *m/z* 931.2188) one of them occurred in the oxidized form (hexuronic acid) (Figure S10). Finally, five tricetin-derived metabolites were detected. They were well separated in RP chromatography, due to the peculiar substitution pattern, which was recognized as dihexuronidyl (**26**), dihexuronidyl dihexosyl (**33**), hexuronidyl dihexosyl *p*-coumaroyl (**34**), dihexosyl *p*-coumaroyl (**54**) and hexosyl *p*-coumaroyl (**62**) moiety (Figure S11).

### 2.2. Relative Quantitation of Polyphenols in Lavandula austroapennina Organs

**Figure 6:**
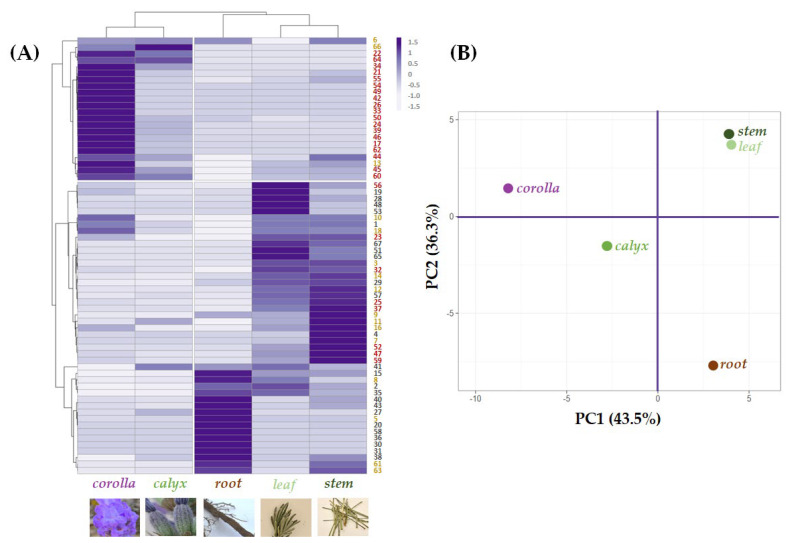
(**A**) Heatmap and (**B**) principal component analysis of tentatively identified compounds—● phenylpropenoyl derivatives; ● phenylpropanoyl derivatives; ● flavonoids—in the five alcoholic extracts from *L. austroapennina* organs.

A multivariate analysis approach was carried out to explore and clarify the relative quantitation of the tentatively identified compounds in each organ, highlighting cluster segregation occurrence (Figure 6A).

Leaf and stem grouped together due to their abundance in compounds based on (or deriving from) hydroxycinnamoyl skeleton or its 8-hydroxydehydro derivative. Yunnaneic acid D isomers (**30**, **31** and **36**) were in the root, which also appeared rich in nepetoidins **61** and **63**. Esculin (**5**) content further distinguished root extract. Indeed, in the data set about chemical composition profiles of the investigated extracts, the leaf and stem composition was close to each other in the Principal Component Analysis (PCA) score plot of the first two PCs. This provides a map of how the organs relate to each other based on their (poly)phenolic content. In fact, the first component, PC1, which accounts for 43.5% of the variation, allowed corolla and calyx, with a higher abundance of flavonoids, to be positioned in the negative score, while the other organs, characterized by a lower content in these constituents, were at the end of the positive axis. In addition, the second component, PC2, reaching 36.3% of the variance, was responsible for a further separation of the organ groups of both quadrants. In particular concerning to corolla and calyx, although both of these epigeal organs are mainly made up of flavonoids, the corolla is distinguished not only by the higher relative abundance of these compounds but also by the exclusive presence of glycosylated acylated flavonoids. The latter is characterized by having a glyconic portion with also hexuronic acid residues. A coumaroyl moiety characterized the acyl moiety. Regarding the root organ, it was located at the end of the negative score of the PC2 axis, while the leaf and stem in the positive one due to the different chemical compositions described at the beginning of this paragraph.

### 2.3. Antioxidant Activity of Lavandula austroapennina Alcoholic Extracts

Data from the in vitro antiradical capability assessment of the alcoholic extracts from *L. austroapennina* organs were preliminarily analyzed by cluster analysis, to explore the degree of dissimilarity values between test types and plant organs.

**Figure 7:**
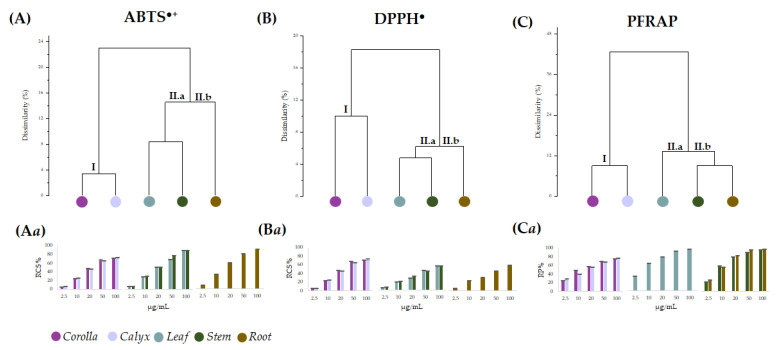
Dendrograms based on data from antioxidant assays ABTS (**A**), DPPH (**B**) and PFRAP (**C**) carried out using the alcoholic extract of the different *Lavandula austroapennina* organs. Radical scavenging capacity (RSC, %) of values expressed as mean ± SD following ABTS method (**Aa**) and DPPH method (**Ba**). Reducing Power (RP%) data are in (**Ca**). All the data are expressed as mean ± SD of three experiments, independently carried out, each of which in triplicate.

An average linkage agglomeration criterion and Jaccard Index as dissimilarity coefficient were applied to each (6 organs × 5 concentrations) of the data matrix from radical scavenging activity (ABTS^•+^, and DPPH^•^) and reducing power (PFRAP). The obtained dendrograms (Figure 7A–C) distinctly displayed different clustering patterns based on the applied antioxidant tests. The dendrogram through ABTS data assay highlighted two main clusters, with a dissimilarity value of 24%. According to the chemical composition results, the first cluster contained corolla and calyx (Figure 7A_(I)_), while the second consisted of two subclusters, including leaf and stem (Figure 7A_(II.a)_) on one side and root on the other (Figure 7A_(II.b)_). An equal correlation was found considering DPPH assay data, whereas PFRAP assay data distinguished leaf from stem and root as subgroups of cluster II (Figure 7C_(II.a,II.b)_). Based on the cluster analysis outcome, antioxidant activity data were organized accordingly (Figure 7Aa–Ca). It is evident that for all the assays, the values of leaf, stem and root extracts were grouped into a single cluster, which involved two subclusters sharing the same actors for both the antiradical tests, while leaf extract occupied alone subcluster C_II.a_ in PFRAP dataset. Corolla and calyx were always in cluster I and showed a similar concentration-dependent trend, resulting in the organs with the least bioactivity at the lowest doses tested. Moreover, leaving aside the response observed in the DPPH assay, it appears that all the organs exerted a strong antioxidant efficacy at the highest concentrations (from 25 to 100 μg/mL). In fact, the data were pooled with decreasing activity from corolla and calyx (cluster I) to leaf, stem and root (cluster II). The marked antioxidant efficacy of leaf, stem and root extracts could be due to their diversity in HCA-derived polyphenols showing free catechol moieties, able to easily transfer two electrons and their poor content in flavonoid glycosides, which were abundant in corolla and calyx extracts [51,52,53]. In fact, glycosylation appears to impact negatively the antioxidant capability [54]. Indeed, saccharidic moieties linkage to flavonoids affects also their rates of absorption and metabolization, and it was suggested that the effects of glycosylation on flavonoid bioactivity in vitro may differ from that observed in vivo [55].

In the literature, there are various data relating to the antioxidant efficacy of different cultivars of *Lavandula angustifolia* and other species such as *L. hybrida* or *L. viridis*, although, for the most part, the investigation is limited to the aerial portions of the species [56].

### 2.4. Cytotoxic Screening of Alcoholic Extract from Lavandula austroapennina Organs

**Figure 8:**
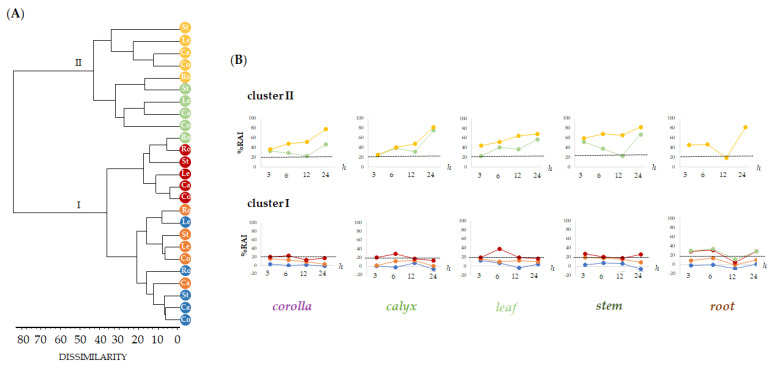
Dendrogram of cytotoxic activity by MTT test carried out on the alcoholic extracts of different *Lavandula austroapennina* organs (Co, Corolla; Ca, Calyx; Le, Leaf; St, Stem; Ro, Root) at increasing doses (● 1, ● 5, ● 10, ● 50 and ● 100 μg/mL). (**A**). Redox activity inhibition (%RAI) of each organ and different exposure times (3, 6, 12 and 24 h) ordered as depicted by dendrogram (**B**). RAI, Redox activity inhibition: values are expressed as mean ± SD of two independent experiments, each of which in six replicates.

The HaCaT human keratinocyte cell line was used to preliminarily evaluate by means of MTT assay the cytotoxicity of alcoholic extracts from *L. austroapennina* organs, using a broad range of dose levels (from 1 µg/mL to 100 µg/mL), at different exposure times (3, 6, 12 and 24 h). Human keratinocytes are a valid, in vitro, model for studying the toxicity profile of botanical products, as well as the inflammatory response, the healing properties and also for assuring dermoprotection against ROS-induced stress [57,58,59,60]. Data obtained, analyzed as the mean of three replicates, were organized into data matrix (5 organs × 5 concentrations × 4 treatment times) and processed by cluster analysis to explore the dissimilarity degree between plant organs, tested concentrations and treatment times. An agglomeration criterion of mean linkage and the Jaccard index as a coefficient of dissimilarity were applied. The obtained dendrograms (Figure 8A) highlighted the different clustering pattern of the dose levels tested in relation to the organs with two main clusters reaching a dissimilarity value of 80%.

The first group (cluster I) included concentrations able to reduce mitochondrial cell viability to less than 25% (1, 5, 10 μg/mL), while the second cluster grouped dose levels at 50 and 100 μg/mL, which showed higher %RAI (cluster II). Based on clustering, data were plotted to highlight the cytotoxicity trends in relation to the tested extract concentrations and exposure time (Figure 8B). This is with the only exception of leaf extract, which showed an inhibition of mitochondrial redox activity equal to 38.4% after 6 h of treatment time. Cluster II represented the tested 50 and 100 μg/mL dose levels, whose higher cytotoxic activity occurred as exposure time rises. Notably, the root alcoholic sample was the only one whose bioactivity at 50 μg/mL disclosed a redox activity inhibition equal to 12.32%. Exceptionally, cytotoxicity decrease was remarkable after 12 h (corolla, RAI_%_ = 22.87 ± 0.40; calyx, RAI_%_ = 31.95 ± 0.21; stem, RAI_%_ = 22.65 ± 0.67). Data acquired reveal that 12 h of time treatment may be the optimum to allow the bioactivity of specialized metabolites involved in each extract to provide maximum skin health benefits.

### 2.5. Alcoholic Extracts from Lavandula austroapennina Exert Wound-Healing Activity

**Figure 9:**
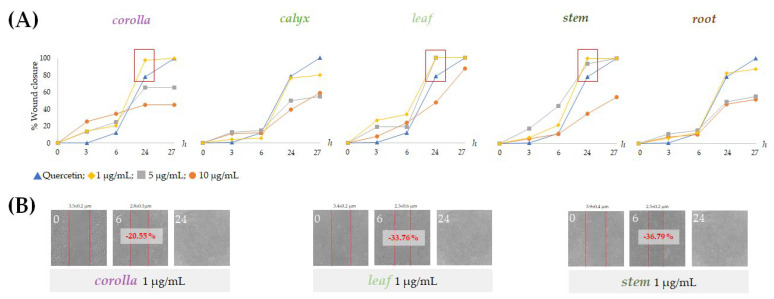
Scratch wound assays carried out on HaCaT cells. (**A**) Wound closure % reported as mean ± SD from two independent measurements, each carried out in triplicate and (**B**) representative wounded cells at 0, 6 and 24 h after wounding.

The effects of non-cytotoxic doses of the prepared extracts from *L. austroapennina* on skin cell migration were evaluated by in vitro analysis of the scratch wound on HaCaT cell monolayers. The ability of HaCaT keratinocytes to migrate closing the wound allowed the evaluation of the healing process by measuring the wound width immediately after the wound and at considered time intervals. The extracts from corolla, leaf and stem were particularly active at low doses. In fact, they induced rapid wound closure on HaCaT cells at a concentration of 1 µg/mL (Figure 9B). In particular, stem extract provided a closure equal to −36.8% after 6 h. This could be due to its being a mixture of small hydroxycinnamic acid derivatives, such as compounds **7**, **9**, **11**, **16** and flavonoid glycosides.

Indeed, hydroxycinnamic acid derivatives are intensively explored in pharmaceutical, biomedical, nutraceutical and cosmeceutical fields due to their broad spectrum of activity resulting from their chemical structure. The benzene ring and acrylic acid residue enable various chemical modifications with improved bioactive properties, enhanced electron withdrawn ability, modified lipophilicity and improved absorption and biodistribution [61,62]. These compounds have also been widely used in cosmetics for their UV-absorbing and filtering properties, antioxidant, or for skin/hair conditioning, or also as antimicrobial ingredients [63]. In addition, they have been investigated for their depigmenting activity onto a model of UVB-induced hyperpigmentation via tyrosinase inhibition, thus acting as a skin-whitening agent [64]. Moreover, they could act at different levels in the wound-healing steps, even in chronic situations. Ghaisas et al. [65] evaluated the involvement of HCAs activity on specific molecular targets, such as NO (nitric oxide), SOD (superoxide dismutase), glutathione (GSH), hydroxyproline and hexosamine, being efficacious in reducing inflammation, activating antioxidant pathways and promoting new skin tissue formation. Ferulic acid proved to be active in an induced AD mouse model, reducing the expression of cytokines, such as IL-4, IL-6, TNF-α and IL-31, and suppressing the immune-mediated response of T helper type 2 (Th2) cells [66]. In addition, the caffeic acid derivative evidenced high anti-inflammatory activity in atopic dermatitis by suppressing pro-inflammatory cytokines and NF-κB protein production in keratinocyte cells [67]. Rosmarinic acid was mostly investigated for psoriasis chronic skin disorder. Its topic administration was demonstrated to statistically reduce levels of IL-6, IL-8, TNF-α and NF-κB involved in inflammatory response of epidermal keratinocytes [68]. *Lavandula angustifolia* Mill. extract, rich in rosmarinic acid, proved to interfere the JAK1/STAT2 signaling pathway, inducing downregulation of NF-κB gene expression and affecting the PI3K/AKT signaling, engaged in the psoriasis condition [69]. Among HCA derivatives, salvianolic acids are reported to be stronger antioxidant than other phenolic compounds [32]. Salvianolic acid B is reported to have pro-angiogenesis, antiapoptosis and antioxidative stress effects by stimulating autophagy that enhances the survival of skin flaps and wound healing [70]. Guo et al. [71] suggested that salvianolic acid B-microemulsion formulation could be a good candidate for topical antipsoriasis treatment by reducing inflammatory response through down-regulating IL-23/IL-17 pathway, inhibiting abnormal proliferation of keratinocytes and moisturizing dry skin. In addition, salvianolic acid B and danshensu elicited proliferative activity for Detroit 551 fibroblast cells as well as the ability to increase collagen (type I and V) production, likely through the activation of the TGF-β/Smads fibroblast signaling pathway. These compounds may also be able to suppress melanin production by inhibiting the enzyme tyrosinase. Furthermore, they could potentially be used as an agent for the treatment of hyperpigmentation and wound healing [72]. The role of flavonoid compounds should also be considered, so that flavonoid aglycones are components of various pharmaceutical, medical and cosmetic applications due to their antioxidative, anti-inflammatory, antimutagenic and antiaging properties [73,74]. Indeed, the bioactivity of flavonoids depends on the arrangement of functional groups around the core structure and appears to be markedly affected by glycosylation, which confers decreasing activity, as well as the number of glyconic moieties is related to the decrease in both compound lipophilicity and transdermal bioavailability [75].

## 3. Materials and Methods

### 3.1. Plant Collection and Extraction

*Lavandula austroapennina* plants were collected in July 2021 from Mt. Cervati (Sanza Municipality, 40°15′19.6″ N 15°28′42.8″ E, 11,801,250 m asl) in the Cilento, Vallo di Diano and Alburni National Park (Southern Italy). Taxonomic identification was performed following Pignatti et al. [76,77]. A voucher specimen has been deposited in the Herbarium Austroitalicum (IT, acronym follow Thiers 2023 [78]) of the University of Campania Luigi Vanvitelli (Caserta, Italy).

**Figure 10:**
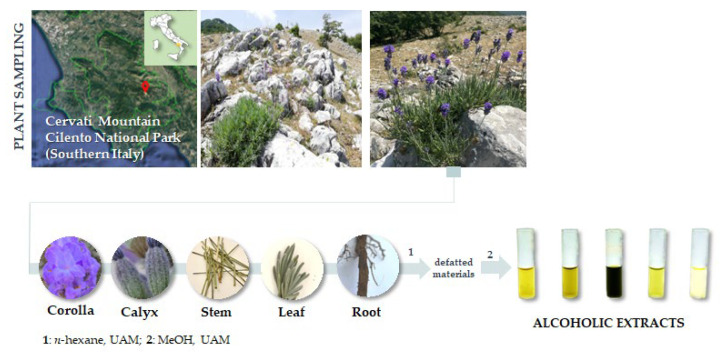
*Lavandula austroapennina* sampling site and extraction scheme. UAM = Ultrasound-Assisted Maceration.

After harvesting, each plant material was in situ divided into corolla, calyx, leaf, stem and root, then marked and immediately stored in liquid nitrogen. Each plant organ was first lyophilized and pulverized by a rotating knife homogenizer (Knife Mill PULVERISETTE 11, Buch & Holm, Herlev, Denmark). Dried material underwent sequential extraction by ultrasound-assisted maceration (UAM; Branson Ultrasonics^TM^ Bransonic^TM^ M3800-E; Danbury, CT, USA) using sequentially *n*-hexane and methanol as extractive solvents in a plant matrix:solvent ratio as 1:20 (g plant matrix:mL solvent). Three UAM cycles by each solvent were carried out (30 min each; Figure 10).

### 3.2. UHPLC-ESI-QqTOF-MS and MS/MS Analyses

The methanolic extract was investigated using the NEXERA UHPLC system (Shimadzu, Tokyo, Japan) equipped with a Luna^®^ Omega C-18 column (50 × 2.1 mm i.d., 1.6 μm particle size). Two μL of each sample were injected. The mobile phase was constituted by water (solvent A) and acetonitrile (solvent B), both acidified with formic acid (0.1% *v/v*). A linear gradient was used as follows: 0–10 min, 5%→32% B; 10–28 min, 32→75% B; 28-29 min, 75%→95% B; 29–30 min, 95% B; 30–32 min, column re-equilibration. The flow rate was set at 400 μL/min. High-Resolution Mass Spectrometry (HR-MS) data were obtained by an AB SCIEX Triple TOF^®^ 4600 mass spectrometer (AB Sciex, Concord, ON, Canada), equipped with a DuoSpray^TM^ ion source (AB Sciex, Concord, ON, Canada) operating in the negative ElectroSpray (ESI) mode. A full-scan Time-Of-Flight (TOF) survey and 8 information-dependent acquisition MS/MS scans were acquired, using the following parameters: curtain gas 35 psi, nebulizer and heated gases 60 psi, ion spray voltage 4500 V, ion source temperature 600 °C, declustering potential −80 V and collision energy −40 ± 15 V. The instrument was controlled by Analyst^®^ TF 1.7 software (AB Sciex, Concord, ON, Canada), whereas MS data were processed by PeakView^®^ software version 2.2 (AB Sciex, Concord, ON, Canada).

### 3.3. Antioxidant Assessment

The alcoholic extracts from *L. austroapennina* organs were tested at 2.5, 10, 20, 50 and 100 μg/mL towards the ABTS [2,2′-azinobis-(3-ethylbenzothiazolin-6-sulfonic acid)] radical cation and 2,2-diphenyl-1-picrylhydrazyl (DPPH) radical.

The previously prepared ABTS^•+^ solution was diluted in phosphate buffer saline (PBS; pH 7.4) to achieve an absorbance of 0.7 recorded at 734 nm [79] and added to extracts in order to achieve the final tested dose levels. The absorbance values were taken after 6 min by a Victor3 spectrophotometer (Perkin Elmer/Wallac; Waltham, MA, USA) and plotted vs. the blank. The DPPH^•^ free radical scavenging capacity was also evaluated as previously described [79], and absorbances were recorded at 517 nm. Three replicate measurements for each sample (three for each concentration) were performed.

The potassium ferricyanide reducing power (PFRAP) assay was also performed to estimate the reducing power of the investigated methanolic extracts (2.5, 10, 20, 50 and 100 μg/mL; final concentration levels). The absorbance was measured at 700 nm [79]. A blank was considered, preparing a solution with PFRAP reagent without samples. All data were expressed as mean ± standard deviation (SD).

### 3.4. Cell Culture and Cytotoxic Screening

Human primary keratinocytes cell lines (HaCaT) were cultured in Dulbecco’s Modified Eagle’s Medium (DMEM) supplemented with 10% fetal bovine serum, 50.0 U/mL of penicillin and 100.0 μg/mL of streptomycin, at 37 °C in a humidified atmosphere containing 5% CO_2_. Cells were seeded in 96-multiwell plates at a density of 1.5 × 10^4^ cells/well and were treated with the organ polar extracts at 1, 5, 10, 50 and 100 μg/mL, at different treatment times (3, 6, 12 and 24 h). Then, the inhibition of mitochondrial redox activity (RAI %) was determined with the MTT cell test [79]. Two independent experiments were carried out with six replicate measurements for each concentration of each extract. Data were expressed as mean ± standard deviation (SD).

### 3.5. Wound Scratch Assay

HaCaT cells (5 × 10^5^) were seeded in a 60-mm dish. After 24 h, as a confluent monolayer was reached, a wound was simulated by manually scraping the cell monolayer with a p200-pipette tip, and the cell layer was washed three times using PBS (1 mL × 3). Cells were thus treated with non-cytotoxic dose levels of the extracts (1, 5 and 10 µg/mL); quercetin 10 µM was used as a positive control, while cells treated only with culture medium served as a negative control. The initial wound quantification was performed on images collected 3 h after wounding when the wound size had stabilized. Additional images were collected at 6, 12, 24 and 48 h after wounding. Wound healing over time was calculated manually using Photoshop 2008 applying the following proportion: FOV (Microscope Field of View) × Size (Photoshop)/Diameter (Photoshop) [80]. The FOV value was obtained from the NIKON TE300 microscope datasheet source. The data, which were from two independent measurements, each one in triplicate, were reprocessed as %Wound closure [81].

### 3.6. Statistical Analyses

A multivariate analysis approach by ClustVis (https://biit.cs.ut.ee/clustvis/, accessed on 15 September 2022) was adopted to explore and clarify quali-quantitative compositive data of compounds, as acquired by UHPLC-QqTOF-ESI-MS analysis, in each organ. Numerical clustering of antioxidant (DPPH, ABTS and PFRAP) and cytotoxic (MTT) assay data was made on the basis of mean values of three and six replicates for each of the five extract concentrations tested for each of the five *L. austroapennina* organs (corolla, calyx, stems, leaf, stem and root), using the SYN-TAX software (SYN-TAX 2000, Syntax, Berlin, Germany) [82].

## 4. Conclusions

A systematic analysis aimed at getting insights into the chemistry of each organ of *L. austroapennina* has been carried out. The UHPLC-Q*q*TOF-MS/MS analyses have highlighted the specific distribution of (poly)phenolic compounds in the different organs of corolla, calyx, leaf, stem and root. The diversity in both glycosylated and acylated flavonoids in the corolla contrasts with the high presence of derivatives of hydroxycinnamic acids (e.g., in the stem) and the 8-hydroxy phenylpropanoic acid. Salvianolic acids and yunnaneic acids are highly present in hypogeal organs. The data underline a richness in polyphenolic bioactive metabolites that make each organ a potential resource highly exploitable in the health field. Considering the past use in cosmetic sector, the deepening of the antiradical and reducing effectiveness of mixtures from each organ, as well as the definition of the cytotoxicity profile in keratinocyte cells, lays the foundation for evaluating the positive response in wound-healing assay. Beyond the corolla, which has found employment for obtaining essential oils, parts such as leaf and stem show promising activities that suggest further investigations for the full recovery of this local good.

## Figures and Tables

**Table 1:** TOF-MS and MS/MS data of phenylpropenoic acid derivatives tentatively identified in polar extracts from the different *Lavandula austroapennina* organs. Peak numbers are based on elution order in the whole reversed-phase chromatograms. RDB = ring and double-bond value. Base peaks are labeled in bold.

Peak	Rt	Tentative Assignment	Formula	[M-H]^−^ Found (*m/z*)	[M-H]^−^ calcd. (*m/z*)	Error (ppm)	RDB	MS/MS Fragment Ions (*m/z*)
**3**	2.083	Caftaric acid	C_13_H_12_O_9_	311.0407 623.0903 ^a^	311.0409	−0.5	8	179.0348; 149.0094; **135.0451**
**5**	3.471	Esculin	C_15_H_16_O_9_	339.0716	339.0722	−1.6	8	**177.0188**; 133.0302
**6**	3.886	*p*-Coumaric acid hexoside (isomer 1)	C_15_H_18_O_8_	325.0929	325.0929	0.0	7	163.0405; **119.0507**
**7**	4.350	Fertaric acid	C_14_H_14_O_9_	325.0565	325.0565	0.0	8	193.0511; 178.0267; 149.0610; **134.0378**; 119.0504
**8**	4.360	Caffeic acid hexoside (isomer 1)	C_15_H_18_O_9_	341.0872	341.0878	−1.8	7	179.0356; 161.0246; **135.0457**; 134.0371
**9**	5.525	*p*-Coumaric acid dihexoside	C_21_H_18_O_13_	487.1467	487.1457	2.0	8	487.14621; 163.0394; **119.0501**; 113.0242
**10**	6.164	Ferulic acid hexoside (isomer 1)	C_16_H_20_O_9_	355.1032	355.1035	0.4	7	193.0508; **149.0613**; 134.0377; 133.0294
**11**	6.535	Caffeoylmalic acid (isomer 1)	C_13_H_12_O_8_	295.0462	295.0459	0.9	8	179.0348; 135.0454; 134.0373; **133.0143**; 115.0042; 107.0510
**12**	6.791	Caffeoylmalic acid (isomer 2)	C_13_H_12_O_8_	295.0463	295.0459	1.2	8	179.0349; 135.0453; 134.0370; 133.0145; 115.0043
**13**	7.659	*p*-Coumaric acid hexoside (isomer 2)	C_15_H_18_O_8_	325.0931	325.0929	0.6	7	163.0400; **119.0503**
**14**	7.907	Ferulic acid dihexoside	C_22_H_30_O_14_	517.1572	517.1563	1.8	8	517.1600; **193.0510**; 149.0615; 134.0372
**16**	9.347	Caffeic acid hexoside (isomer 2)	C_15_H_18_O_9_	341.0883	341.0878	1.4	7	179.0345; **135.0450**; 134.0358
**18**	10.021	Ferulic acid hexoside (isomer 2)	C_16_H_20_O_9_	355.1038	355.1035	1.0	7	193.0501; **149.0606**; 134.0373; 133.0297
**61**	19.294	[(Z)-2-(3,5-dihydroxyphenyl)ethenyl] (E)-3-(3,4-dihydroxyphenyl)prop-2-enoate (Nepetoidin A)	C_17_H_14_O_6_	313.0720	313.0718	0.8	11	161.0246; 151.0401; **133.0296**; 123.0438; 105.0345
**63**	19.770	[(Z)-2-(3,4-dihydroxyphenyl)ethenyl] (E)-3-(3,4-dihydroxyphenyl)prop-2-enoate (Nepetoidin B)	C_17_H_14_O_6_	313.0712	313.0718	−1.8	11	161.0243; 151.0400; 150.0318; **133.0292**; 132.0213; 123.0450
**66**	20.861	Tri-*p*-coumaroyl spermidine	C_34_H_37_N_3_O_6_	582.2626	582.2610	2.8	18	582.2651; 462.2058; 436.2262; **342.1446**; 316.1667; 145.0299; 119.0506

^a^ [2M-H]^−^.

**Table 2:** TOF-MS and MS/MS data of phenylpropanoic acid derivatives tentatively identified in polar extracts from the different *Lavandula austroapennina* organs. Peak numbers are based on elution order in the whole reversed-phase chromatograms. RDB = ring and double bond. Base peaks are labeled in bold.

Peak	Rt	Tentative Assignment	Formula	[M-H]^−^ Found (*m/z*)	[M-H]^−^ calcd. (*m/z*)	Error (ppm)	RDB	MS/MS Fragment Ions (*m/z*)
**1**	1.023	8-Hydroxydihydrocaffeic acid (danshensu)	C_9_H_10_O_5_	197.0462	197.0455	3.3	5	179.0357; **135.0450**; 134.0374; 123.0449; 122.0372
**2**	1.699	8-Hydroxydihydrocaffeic acid hexoside	C_15_H_20_O_10_	359.0985	359.0984	0.4	6	359.0986; **197.0456**; 179.0344; 135.0450; 134.0378; 123.0452
**4**	2.22	Dihydrocaffeic acid	C_9_H_10_O_4_	181.0513	181.0506	3.7	5	163.0401; **135.0452**; 134.0376; 119.0501; 117.0353; 107.0499
**15**	7.991	Dihydroferulic acid hexoside	C_16_H_22_O_9_	357.1192	357.1191	0.3	6	195.0661; 177.0553; **151.0766**; 136.0530; 121.0294
**19**	10.060	Yunnaneic acid E	C_27_H_24_O_14_	571.1114	571.1093	3.6	16	527.1224; 483.1327; 439.1417; 329.0678; 285.0777; 241.0878; 215.1082; **197.0461**; 179.0359; 135.0457; 109.0302
**20**	10.325	Lithopermic acid A hexoside	C_33_H_32_O_17_	699.1563	699.1567	−0.5	18	699.1593; 655.1688; 493.1149; 475.1246; 457.1149; 313.0709; **295.0607**; 197.0458
**27**	11.488	Lithospermic acid A	C_27_H_22_O_12_	537.1051	537.1039	2.3	17	537.1062; 493.1169; 313.0729; **295.0626**; 203.0364; 197.0460; 159.0466; 109.0309
**28**	11.656	Yunnaneic acid F (isomer 1)	C_29_H_26_O_14_	597.1257	597.1250	1.2	17	597.1256; 553.1345; 491.1338; 329.1041; **311.0900**; 293.0781; 267.1002; 197.0447; 179.0330; 135.0448
**29**	11.915	Yunnaneic acid F (isomer 2)	C_29_H_26_O_14_	597.1250	597.1250	0.0	17	597.1250; 535.1242; 481.1505; 417.0810; 399.0695; 355.0792; 311.0898; 293.0780; **267.1000**; 241.1198; 197.0435; 179.0332; 135.0437
**30**	12.04	6-(3-(1-carboxy-2-(3,4-dihydroxyphenyl)ethoxy)-3-oxoprop-1-en-1-yl)-3-(3,4-dihydroxyphenyl)-8-hydroxy-7-oxobicyclo [2.2.2]oct-5-ene-2-carboxylic acid (isomer 1)	C_27_H_24_O_12_	539.1204	539.1195	1.7	16	539.1209; 359.0773; 297.0770; 279.0502; 271.0973; 197.0458; 179.0351; **161.0246**; 135.0454; 133.0296
**31**	12.251	6-(3-(1-carboxy-2-(3,4-dihydroxyphenyl)ethoxy)-3-oxoprop-1-en-1-yl)-3-(3,4-dihydroxyphenyl)-8-hydroxy-7-oxobicyclo [2.2.2]oct-5-ene-2-carboxylic acid (isomer 2)	C_27_H_24_O_12_	539.1205	539.1195	1.9	16	539.1227; 359.0783; 341.0666; 315.0884; 297.0778; 271.0982; 253.0876; 135.0772; 197.0463; 179.0357; 161.0250; **135.0461**
**35**	12.903	Rosmarinic acid hexoside	C_24_H_26_O_13_	521.1322	521.1301	4.1	12	521.1333; 359.0782; 323.0775; 197.0460; 179.0353; **161.0248**; 135.0454
**36**	12.979	6-(3-(1-carboxy-2-(3,4-dihydroxyphenyl)ethoxy)-3-oxoprop-1-en-1-yl)-3-(3,4-dihydroxyphenyl)-8-hydroxy-7-oxobicyclo [2.2.2]oct-5-ene-2-carboxylic acid (isomer 3)	C_27_H_24_O_12_	539.1216	539.1195	3.9	16	539.0781; **297.0778**; 279.0665; 197.0460; 179.0355; 161.0250; 135.0455
**38**	13.293	Rosmarinic acid isomer	C_18_H_16_O_8_	359.0775	359.0772	0.7	11	197.0456; 179.0350; **161.0248**; 135.0450; 133.0296; 123.0449
**40**	13.686	Caffeic acid tetramer hexoside	C_42_H_40_O_21_	879.1991	879.1989	0.2	23	879.2054; 835.2154; 699.1620; **681.1519**; 655.1727; 637.1608; 519.0959; 501.1071; 483.0963; 457.1167; 321.0401; 295.0621; 197.0462
**41**	13.963	Rosmarinic acid	C_18_H_16_O_8_	359.0769	359.0772	−1.0	11	197.0450; 179.0348; **161.0244**; 135.0450; 133.0294; 123.0450
**43**	14.245	Salvianolic acid B	C_36_H_30_O_16_	717.1487	717.1461	3.6	22	717.1494; 673.1598; 537.1062; 519.0953; 493.1158; 475.1054; 339.0511; 321.0400; **295.0609**; 179.0351; 135.0453
**48**	14.447	Yunnaneic acid E derivative 1	C_26_H_22_O_12_	525.1054	525.1039	3.0	16	507.0953; **327.0507**; 309.0395; 283.0612; 257.0803; 239.0705; 211.0758; 179.0346; 135.0452
**51**	14.867	Yunnaneic acid F derivative	C_29_H_24_O_13_	579.1157	579.1144	2.2	18	579.1161; 491.1359; 399.0720; 293.0807; 355.0823; 311.0920; 293.0807; 267.1020; **135.0451**
**53**	15.665	Yunnaneic acid E derivative 2	C_26_H_20_O_12_	523.0882	523.0882	0.0	17	523.0882; 505.0769; 479.0978; 325.0335; **299.0545**; 255.0642; 237.0530; 211.0746; 179.0333; 135.0442
**57**	17.567	Salvianolic acid C	C_26_H_20_O_10_	491.0997	491.0984	2.7	17	491.1012; **311.0563**; 267.0661; 265.0502; 135.0454
**58**	18.267	Sinapoyl-hexosyl-rosmarinic acid	C_35_H_36_O_17_	727.1906	727.1880	3.6	18.0	727.1934; 547.1479; 529.1383; 367.1040; **359.0785**; 323.0772; 179.0355; 161.0250
**65**	20.612	Dihydrosalvianolic acid A	C_26_H_24_O_10_	495.1314	495.1297	3.5	15	495.1329; 315.0886; 297.0775; 271.0975; 197.0451; 179.0349; **135.0455**; 134.0374
**67**	21.596	Salvianolic acid A	C_26_H_22_O_10_	493.1137	493.1140	−0.7	16	493.1149; 313.0711; 269.0808; **135.0453**

**Table 3:** TOF-MS and MS/MS data of flavonoids tentatively identified in polar extracts from the different *Lavandula austroapennina* organs. Peak numbers are based on elution order in the whole reversed-phase chromatograms (RDB = ring and double bond). Base peaks are labeled in bold.

Peak	Rt	Tentative Assignment	Formula	[M-H]^−^ Found (*m/z*)	[M-H]^−^ calcd. (*m/z*)	Error (ppm)	RDB	MS/MS Fragment Ions (*m/z*)
**17**	9.450	Dihexosyl hexuronidyl luteolin	C_33_H_38_O_22_	785.1768	785.1782	−1.8	15	**785.1768**; 665.1341; 623.1254; 503.0831; 461.0719; 447.0917; 327.0521; 285.0405
**21**	10.507	Apigenin di-hexuronide	C_27_H_26_O_17_	621.1097	621.1097	0.1	15	621.1160; 445.0802; **269.0463**; 175.0243; 113.0244
**22**	10.668	Hexosyl hexuronidyl apigenin	C_27_H_28_O_18_	607.1335	607.1305	5.0	14	607.1343; **431.1007**; 269.0457; 113.0246
**23**	10.750	Luteolin di-hexuronide	C_27_H_26_O_16_	637.1074	637.1046	4.3	15	637.1107; 461.0759; **285.0413**
**24**	10.867	Myricetin hexoside	C_21_H_20_O_13_	479.0848	479.0831	3.5	12	479.0841; 317.0305; **316.0214**; 271.0238; 178.9977
**25**	10.988	Luteolin hexuronyl-hexoside	C_27_H_28_O_17_	623.1272	623.1254	2.9	14	623.1269; **447.0938**; 285.0395; 284.0316
**26**	11.295	Dihexuronidyl tricetin	C_27_H_26_O_19_	653.1027	653.0996	4.8	15	653.1049; 477.0699; 343.0495; **301.0359**
**32**	12.784	Luteolin hexuronide (isomer 1)	C_21_H_18_O_12_	461.0736	461.0725	2.3	13	**285.0406**
**33**	12.839	Dihexosyl dihexuronidyl tricetin	C_39_H_46_O_29_	977.2092	977.2052	4.1	17	**977.2052**; 815.1562; 801.1844; 639.1281; 477.0647; 301.0354
**34**	12.898	Dihexosyl *p*-coumaroyl hexuronidyl tricetin	C_42_H_44_O_25_	947.2121	947.2099	2.3	21	**947.2099**; 785.1555; 771.1813; 609.1244; 463.0855; 301.0354
**37**	12.991	Luteolin hexoside	C_21_H_20_O_11_	447.0931	447.0933	−0.4	12	447.0940; **285.0401**; 284.0324
**39**	13.482	Malonylhexosyl hexuronidyl apigenin	C_30_H_30_O_19_	693.1338	693.1309	4.3	16	649.1464; 607.1354; **473.1118**; 431.1012; 269.0450; 113.0240
**42**	13.966	Kaempferol hexoside	C_21_H_20_O_11_	447.0954	447.0933	4.7	12	447.0951; 327.0487; 285.0405; **284.0337**; 255.0302; 227.0357; 151.0024
**44**	14.321	Apigenin hexuronide	C_21_H_18_O_11_	445.0794	445.0776	−1.7	13	**269.0455**; 113.0245
**45**	14.376	Apigenin hexoside	C_21_H_20_O_10_	431.0984	431.0984	0.1	12	**269.0454**; 113.0249
**46**	14.42	Dihexosyl *p*-coumaroyl hexuronidyl luteolin	C_42_H_44_O_24_	931.2188	931.2150	4.1	21	**931.2150**; 769.1641; 593.1320; 447.0951; 285.0416
**47**	14.446	Luteolin hexuronide (isomer 2)	C_21_H_18_O_12_	461.0730	461.0725	1.0	13	**285.0400**
**49**	14.616	Trihexosyl *p*-coumaroyl luteolin	C_39_H_50_O_25_	917.2375	917.2357	1.9	20	917.2357; **755.1865**; 609.1611; 593.1303; 489.1149; 447.0961; 325.0911; 285.0405
**50**	14.745	Tricetin	C_15_H_10_O_7_	301.0352	301.0354	−0.6	11	**301.0352**; 255.0312; 239.0331; 215.0394; 191.0345; 149.0246
**52**	15.394	Luteolin malonyl-hexoside	C_24_H_22_O_14_	533.0947	533.0937	1.9	14	489.1065; **285.0402**; 284.0324
**54**	15.924	Dihexosyl *p*-coumaroyl tricetin	C_36_H_36_O_19_	771.1814	771.1778	4.7	19	771.1778; 609.1245; 463.0889; 301.0354; 300.0283
**55**	16.848	Apigenin acetyl-hexoside (isomer 1)	C_23_H_22_O_11_	473.1100	473.1089	2.3	13	473.1115; 413.0882; **269.0444**; 268.0375
**56**	17.018	Luteolin	C_15_H_10_O_5_	285.0404	285.0405	−0.2	11	**285.0408**; 175.0393; 151.0022; 133.0288
**59**	18.777	Apigenin acetyl-hexoside (isomer 2)	C_23_H_22_O_11_	473.1098	473.1089	1.8	13	473.1106; 269.0441; **268.0369**; 239.0312
**60**	19.033	Apigenin	C_15_H_10_O_5_	269.0458	269.0455	0.9	11	**269.0454**; 151.0028; 117.0341
**62**	19.468	Tricetin *p*-coumaroyl hexoside	C_30_H_26_O_14_	609.1263	609.1250	2.2	18	609.1263; **301.0354**
**64**	20.339	Apigenin *p*-coumaroyl hexoside	C_30_H_26_O_12_	577.1371	577.1352	3.3	18	577.1352; **269.0440**

## Data Availability

Not applicable.

## Supplementary Materials

The supporting information can be downloaded at https://www.mdpi.com/article/10.3390/ijms24098038/s1.
