# Supplementary material for: Lavandula austroapennina (Lamiaceae): Getting Insights into Bioactive Polyphenols of a Rare Italian Endemic Vascular Plant
Source: Int J Mol Sci. 2023 Apr 28;24(9):8038. doi: 10.3390/ijms24098038 (PMC10178519; doi:10.3390/ijms24098038)
Supplement: Supplementary file 1 [file ijms-24-08038-s001.zip › ijms-2303826-supplementary.pdf]

Lavandula austroapennina (Lamiaceae): Getting Insights into Bioactive Polyphenols of a Rare Italian Endemic Vascular Plant

Supplementary Materials:

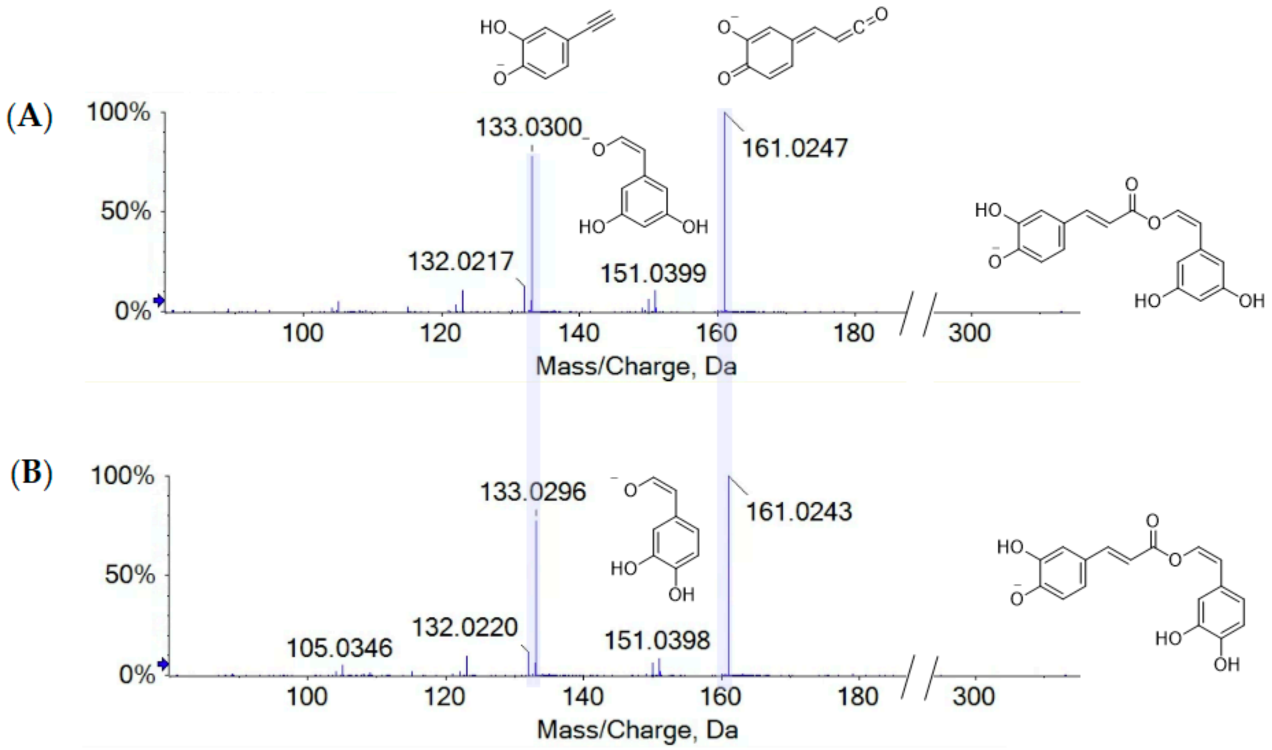

Figure S1. TOF-MS/MS spectra of compound **61** (**A**) and **63** (**B**).

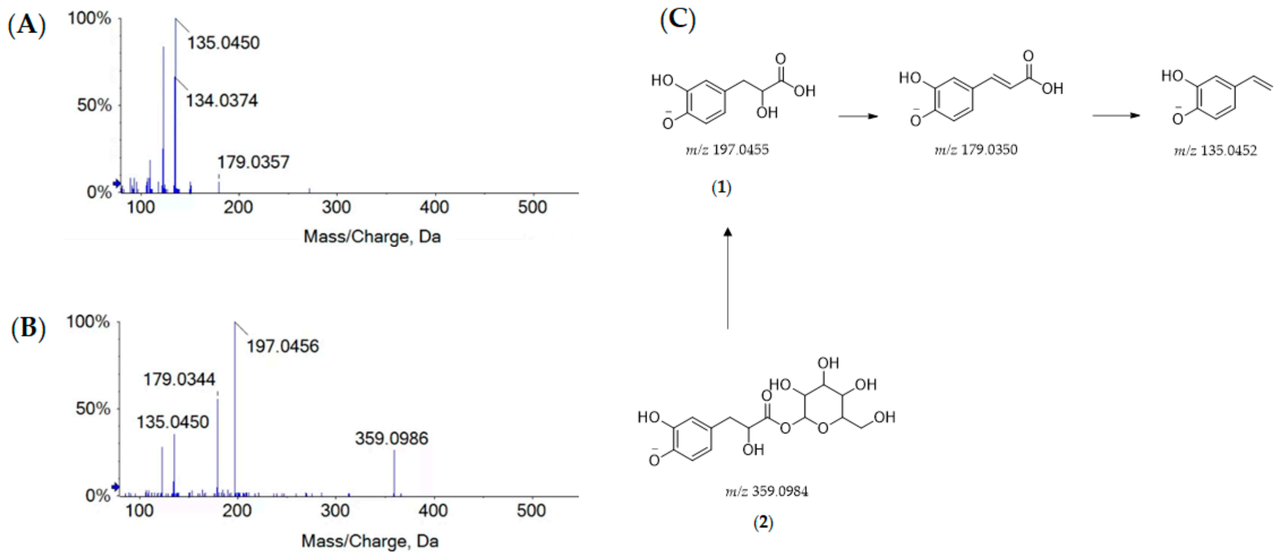

Figure S2. TOF-MS/MS spectra of compound **1** (**A**) and **2** (**B**) and their hypothetical fragmentation (**C**). Theoretical *m*/*z* values are reported below each structure.

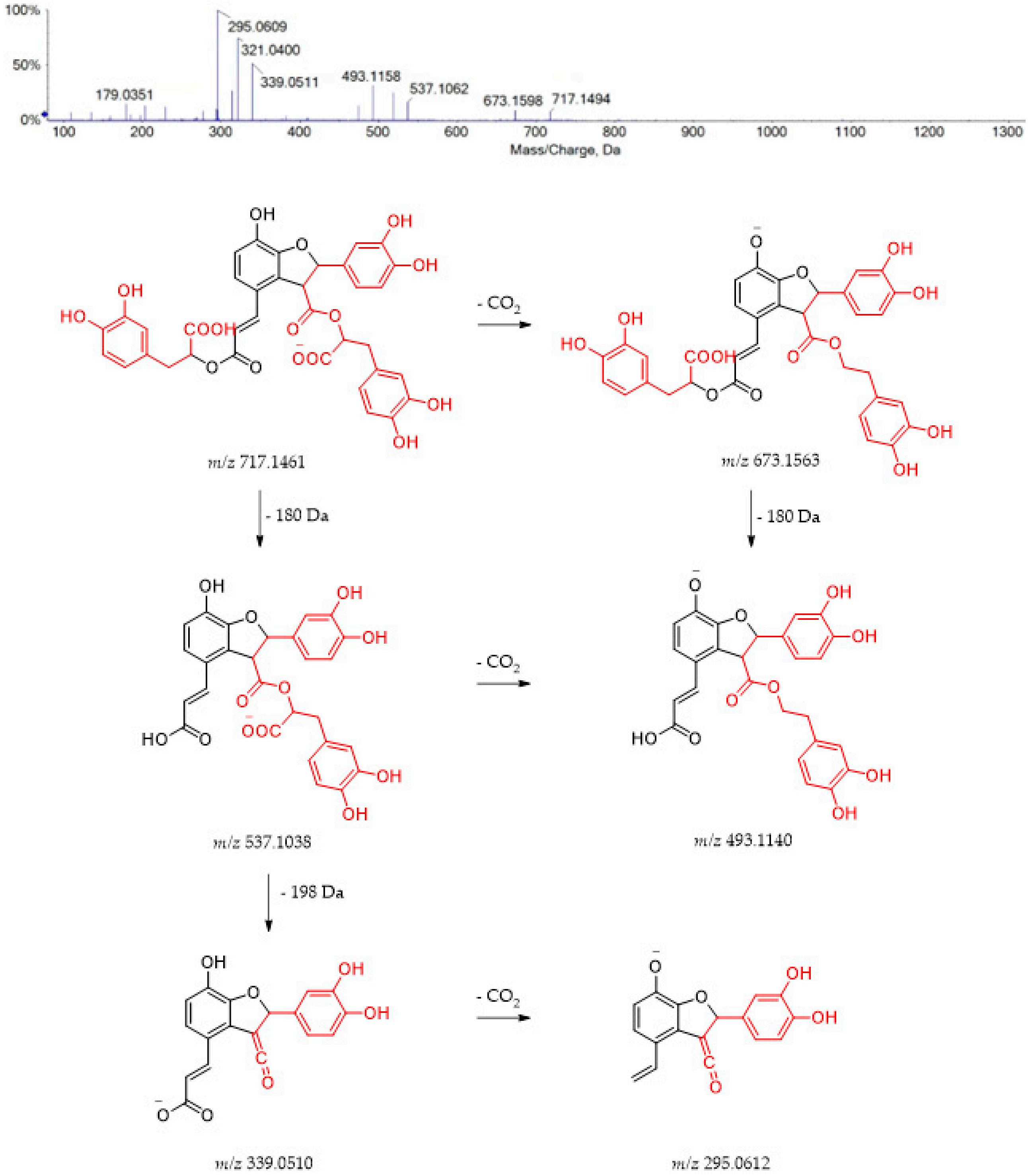

Figure S3. TOF-MS/MS spectrum of salvianolic acid B (**43**) and its hypothesized fragmentation pathway (the three danshensu units are highlighted in red color). Theoretical *m/z* values are reported below each structure.

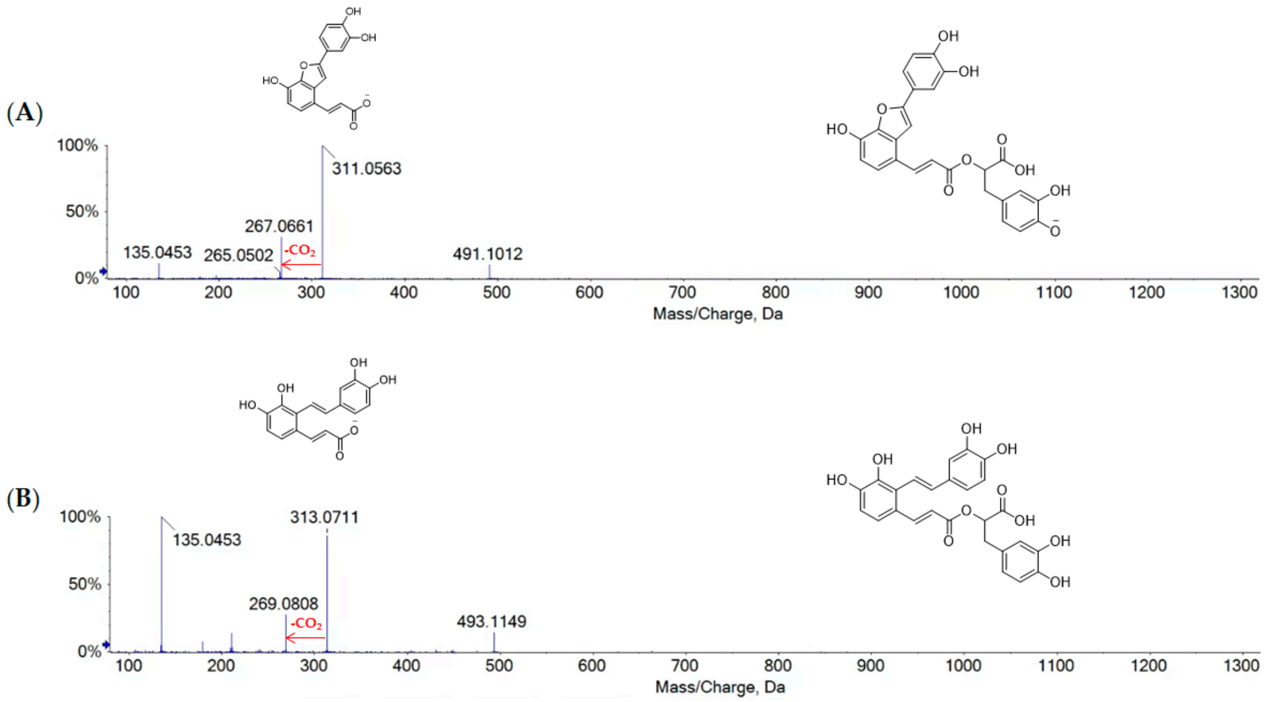

Figure S4. TOF-MS/MS spectra of compounds identified as (**A**) salvianolic acid C (**57**) and (**B**) salvianolic acid A (**67**).

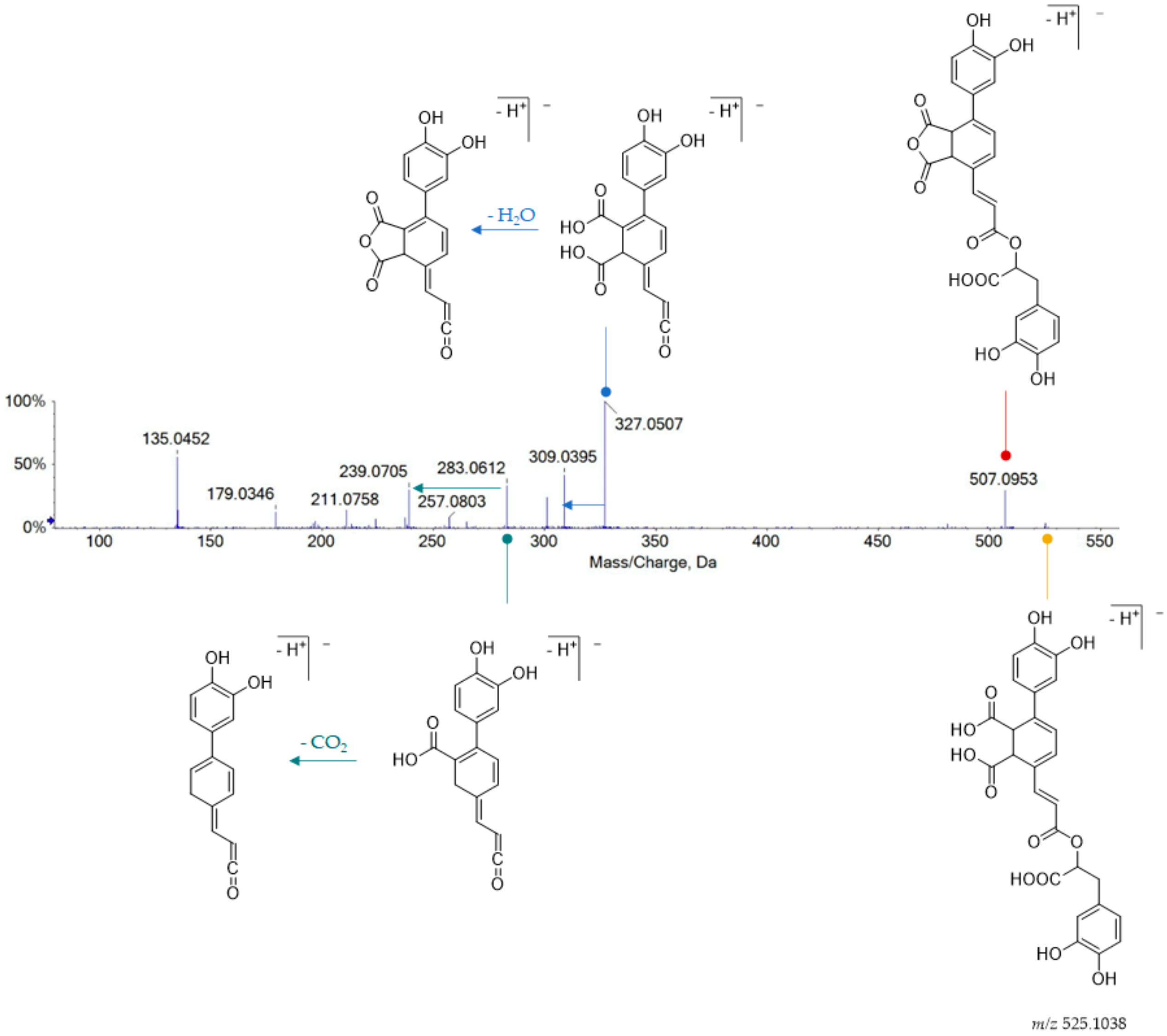

Figure S5. TOF-MS/MS spectrum of compound **48**. The hypothesized pivotal structures are also reported.

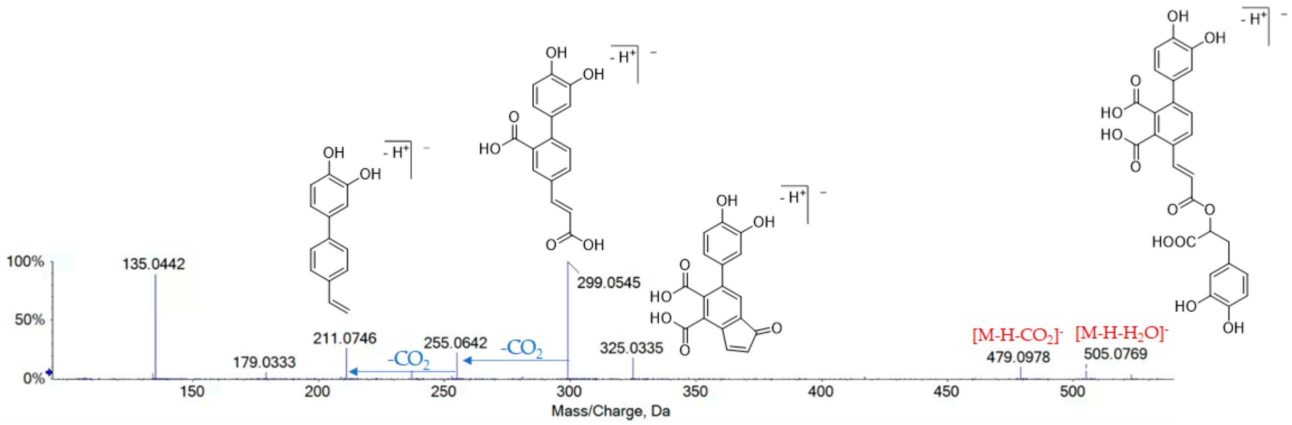

Figure S6. TOF-MS/MS spectrum of compound **53**. The hypothesized pivotal structures are also reported.

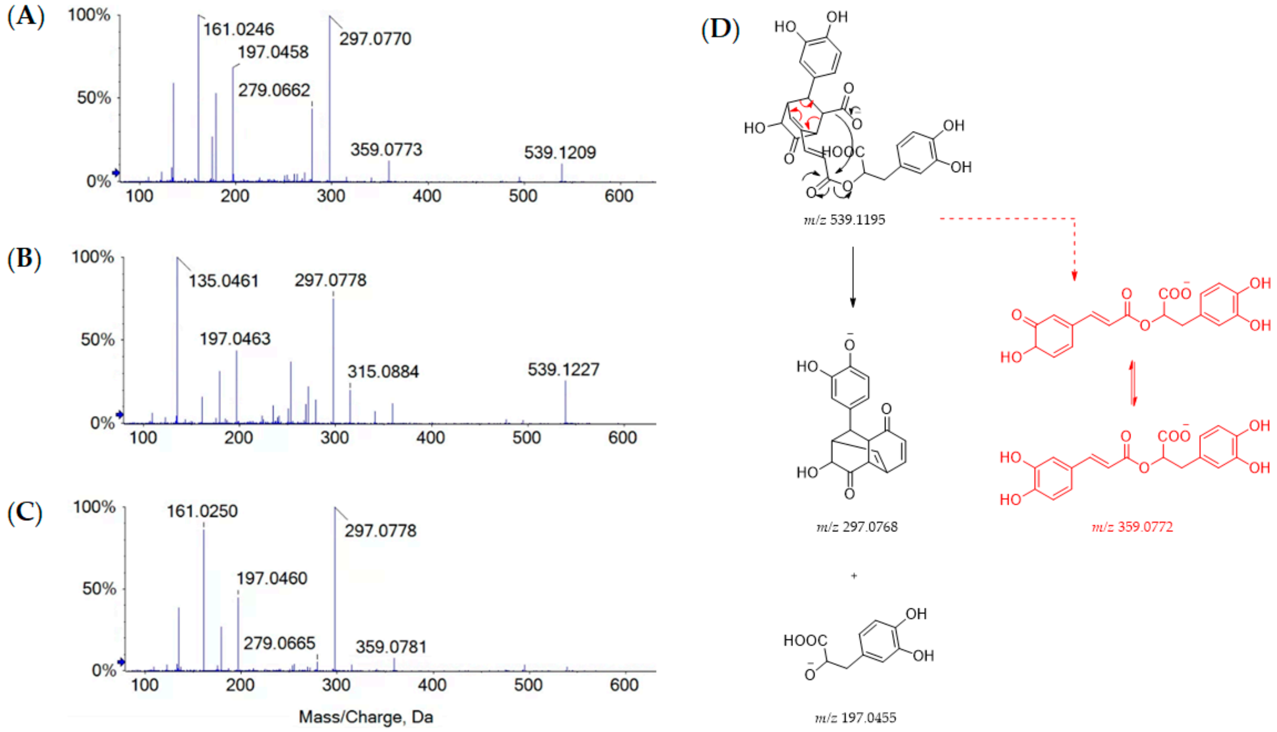

Figure S7. TOF-MS/MS spectra of compounds **30** (**A**), **31** (**B**), and **36** (**C**). The hypothesized pivotal structures are reported (**D**). Theoretical *m/z* values are reported below each structure.

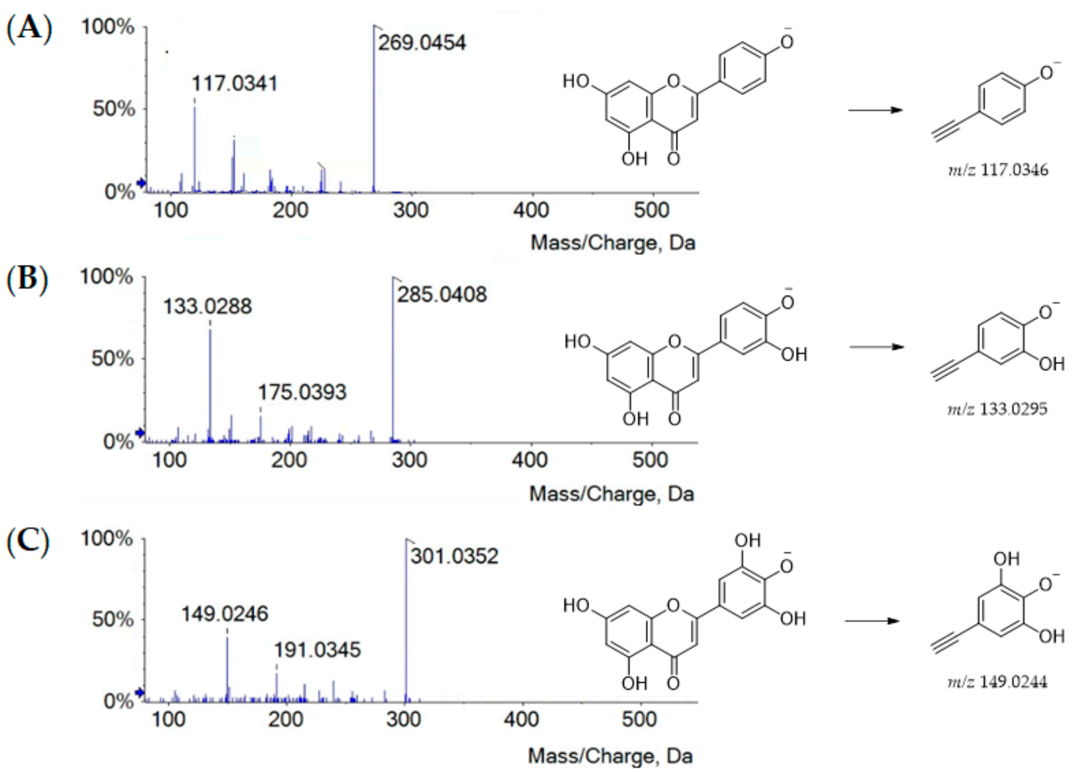

Figure S8. TOF-MS/MS spectra of compounds **60** (**A**), **56** (**B**), and **50** (**C**). The hypothesized pivotal structures are reported. Theoretical *m/z* values are reported below each structure.

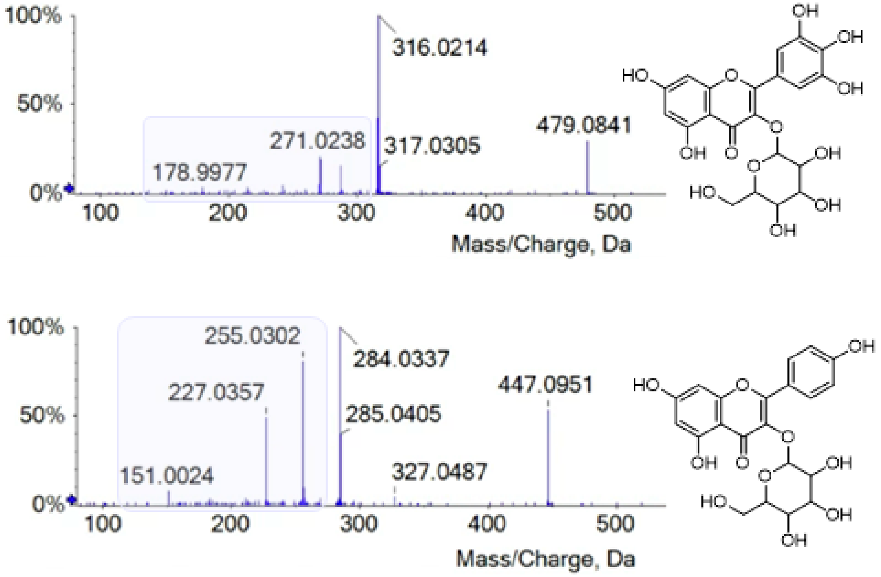

Figure S9. TOF-MS/MS spectra of compounds **24** (at *m/z* 479.0841) and **42** (at *m/z* 447.0951). Coloured boxes denote pivotal ions for flavonol skeleton identification.

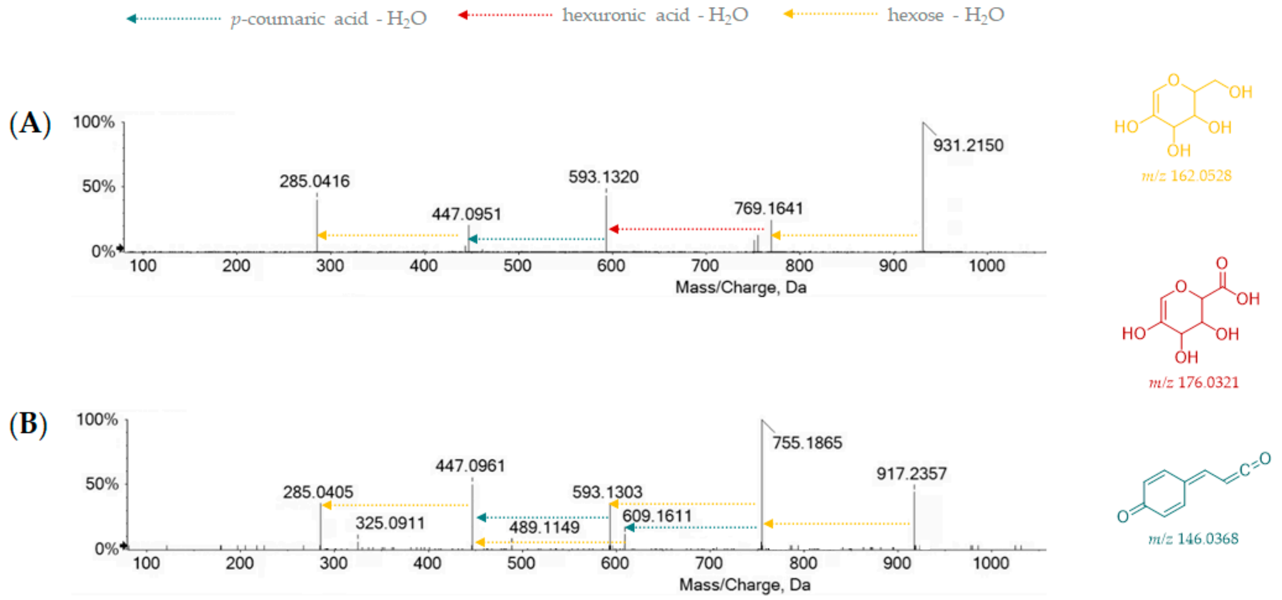

Figure S10. TOF-MS/MS spectra of compounds **46** (**A**), **49** (**B**). Colored arrows highlight the different building blocks of the saccharidic units, sequentially lost as neutral dehydrated form.

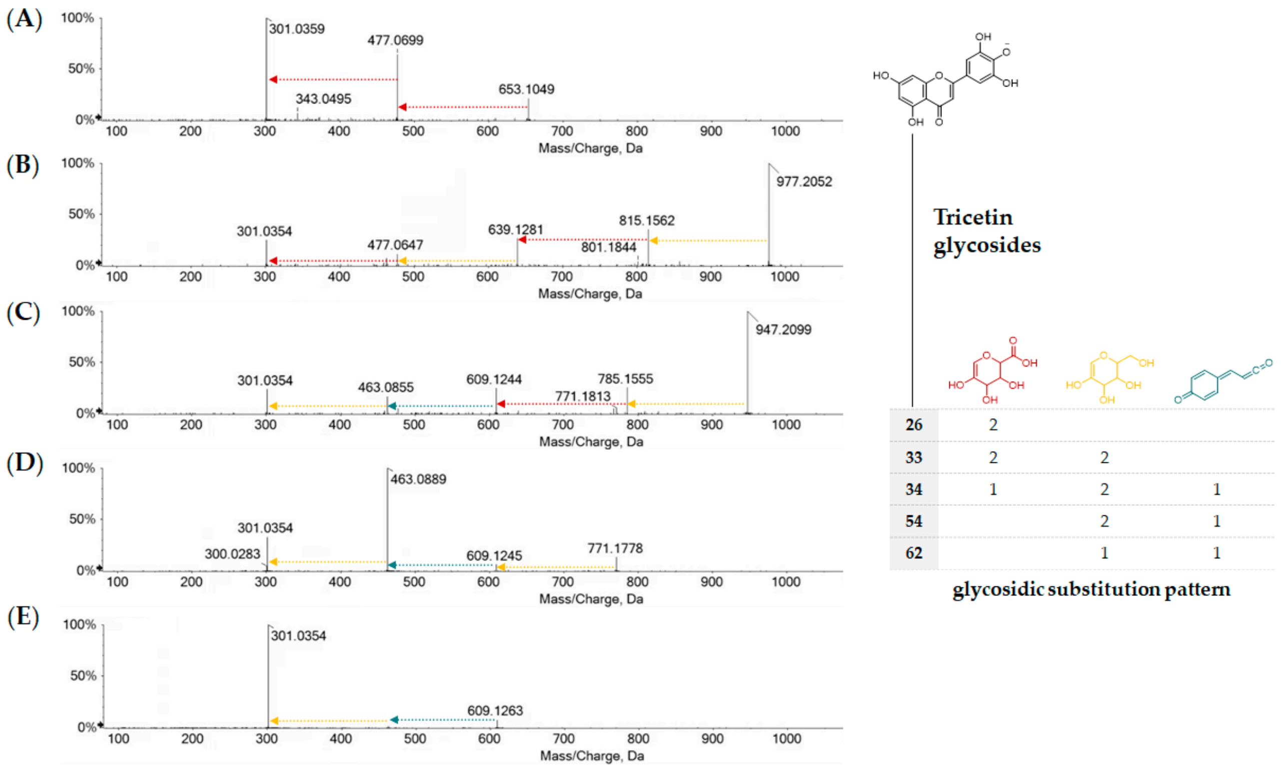

Figure S11. TOF-MS/MS spectra of compounds **26** (**A**), **33** (**B**), **34** (**C**), **54** (**D**), and **62** (**E**). A schematic representation of the glycosidic substitution pattern is also reported.
